# Precise Point Positioning with Partial Ambiguity Fixing

**DOI:** 10.3390/s150613627

**Published:** 2015-06-10

**Authors:** Pan Li, Xiaohong Zhang

**Affiliations:** School of Geodesy and Geomatics, Wuhan University, 129 Luoyu Road, Wuhan 430079, China; E-Mail: lipan.whu@gmail.com

**Keywords:** partial ambiguity resolution, precise point positioning, success rate, ratio test, time to first fix, fixing rate

## Abstract

Reliable and rapid ambiguity resolution (AR) is the key to fast precise point positioning (PPP). We propose a modified partial ambiguity resolution (PAR) method, in which an elevation and standard deviation criterion are first used to remove the low-precision ambiguity estimates for AR. Subsequently the success rate and ratio-test are simultaneously used in an iterative process to increase the possibility of finding a subset of decorrelated ambiguities which can be fixed with high confidence. One can apply the proposed PAR method to try to achieve an ambiguity-fixed solution when full ambiguity resolution (FAR) fails. We validate this method using data from 450 stations during DOY 021 to 027, 2012. Results demonstrate the proposed PAR method can significantly shorten the time to first fix (TTFF) and increase the fixing rate. Compared with FAR, the average TTFF for PAR is reduced by 14.9% for static PPP and 15.1% for kinematic PPP. Besides, using the PAR method, the average fixing rate can be increased from 83.5% to 98.2% for static PPP, from 80.1% to 95.2% for kinematic PPP respectively. Kinematic PPP accuracy with PAR can also be significantly improved, compared to that with FAR, due to a higher fixing rate.

## 1. Introduction

Integer carrier phase ambiguity resolution (AR) is the key to fast and high-precision Global Positioning System (GPS) positioning. For relative positioning, double differenced (DD) carrier phase ambiguities are often fixed to integers in precise GPS applications. Although the accuracy and reliability of estimates benefit much more with more ambiguities correctly fixed, in fact it is not always optimal to fix all the ambiguities because the probability of successfully fixing them decreases and the computational burden increases with increasing number of ambiguities. For this reason, partial ambiguity resolution (PAR) methods were proposed in order to maintain a sufficiently high success rate [1]. The PAR methods for DD ambiguity resolution have attracted the attention of many researchers and have been extensively investigated [2,3,4,5].

Precise point positioning (PPP) [6] is an absolute positioning technology, which can deliver centimeter-level positioning on a global scale with only a single receiver. In recent years, researchers have focused on fixing integer ambiguities for GPS PPP, and several methods have been proposed [7,8,9,10,11,12,13]. PPP AR has become a focus in the GNSS community because of its advantages of accuracy and reliability over a traditional float PPP solution. PPP AR is typically conducted by applying the full ambiguity resolution (FAR) method that fixes all ambiguities. Compared with short-baseline relative positioning, more parameters need to be estimated in PPP and some of the parameters such as ambiguities, receiver clock and zenith tropospheric delay are highly correlated. It is more difficult to fix all ambiguity parameters reliably for PPP than in DD-based relative positioning. Also, typically, a convergence time of several tens of minutes is required to fix ambiguities reliably [14], especially resolving the ambiguities of a newly risen satellite or a re-initializing ambiguity after a cycle slip or data interruption if FAR is used.

Verhagen *et al*. [15] first presented two PAR approaches with different subset selection strategies for PPP AR, where both the requirements on success rate and performance improvement are taken into account. In their study, only a static dataset with simulative perfect orbit and clock corrections is selected to compare the performance, which mainly refers to positioning accuracy, of PPP FAR and PAR. This result may be not representative for real-life PPP application. In addition, the fixing rate and the time to first fix (TTFF) of PPP FAR and PAR are not assessed and compared.

Shi and Gao [16] also applied a PAR method for PPP. They sorted all narrow-lane un-decorrelated ambiguities in the order of ascending estimate precision. They added the candidate with the highest precision from the remaining ambiguities one at a time to determine the subset with maximum ambiguities in an iterative process, validating each subset by applying the success rate and ratio tests. However, this method may result in a subset suffering from poor signal geometry because that ambiguities of high elevation satellites are generally more accurate and likely to be selected. Besides, only preliminary experimental results with one hour of observations from a single station were presented. This may be not convincing and representative for typical performance.

Although attention has begun to be paid to improving the performance of PPP by PAR, there are few convincing and representative studies on what improvement can be brought by PAR, on different aspects of the performance of PPP AR. In order to investigate the effect of PAR on the performance of PPP, we propose a modified PAR method, based on the method of Wang and Feng [5], to find a subset of decorrelated ambiguities which can be fixed with high confidence. First an elevation and standard deviation criterion are used to remove the low-precision ambiguity estimates for AR. Then the success rate and ratio-test are simultaneously used in an iterative process to increase the possibility of finding a potential subset, compared with the method by Wang and Feng. For briefness, we will denote this method as the “WF method”. It is expected to improve the performance of PPP AR, including speeding up the first fixed solution and increasing the fixing rate. For ambiguity-fixed solutions, TTFF is often involved due to the fact that once the ambiguity parameter can be fixed to a correct integer, the positioning accuracy can significantly be improved. Therefore, TTFF is used as an indicator of convergence speed for ambiguity-fixed PPP. The fixing rate is defined as the ratio of the number of fixed epochs to the number of total epochs. Besides, the influence of PAR on the positioning accuracy is also investigated. The data from about 450 globally distributed International GNSS Service (IGS) [17] stations, during DOY 021 to 027 in 2012 are used to test and validate the modified PAR method for PPP.

In the following section, a brief introduction to a traditional PPP FAR method is given, and then a modified PAR procedure with a predefined success rate and ratio threshold is presented in Section 2. Experimental design and data processing are described in Section 3. In Section 4, numerical results are presented that demonstrate the performance of the PAR method for PPP. Finally, in Section 5 the main points and the conclusions are summarized.

## 2. PPP Ambiguity Resolution

In this study, the PPP ambiguity fixing strategy proposed by Ge *et al*. [7] is applied, in which the single differenced (SD) ambiguities between satellites are fixed. First, SD wide-lane and narrow-lane uncalibrated phase delay (UPD) [18] for all satellites are estimated at the server end. Detailed description and analysis of UPD estimation have been well documented by [7,19]. Daily wide-lane UPDs and fifteen-minute narrow-lane UPDs products for all satellites can be generated for PPP users for achieving an accurate and reliable ambiguity-fixing PPP solution. The ambiguity fixing procedure at the PPP-user end is described in the following sections.

### 2.1. Full Ambiguity Resolution for the PPP User

At the PPP user-end, ambiguity resolution can be conducted in two sequential steps. First, the SD wide-lane ambiguities are derived from Melbourne-Wübbena (MW) combination [20,21] and then corrected with satellite wide-lane UPD estimates. The MW combination eliminates the ionospheric delay, tropospheric delay and geometric distance and retains only wide-lane ambiguity with dual frequency observations. The SD wide-lane ambiguities can easily be fixed by rounding averaged wide-lane ambiguities over several epochs. After successfully fixing the wide-lane ambiguities, the narrow-lane ambiguities are derived from the float ionosphere-free ambiguities and the integer wide-lane ambiguities. They can then be fixed after taking the satellite narrow-lane UPD corrections into consideration. Since narrow-lane ambiguities are strongly correlated in PPP, a search strategy based on the LAMBDA method [22] is applied to fix the SD narrow-lane ambiguities. An ionosphere-free ambiguity is fixed only when both its wide- and narrow-lane are fixed. After validation, the remaining real-valued position parameter estimates can be given as:

(1) $$\overset{⌣}{b}=\hat{b}-Q_{\hat{b}\hat{N}}Q_{\hat{N}}^{-1}\left(\hat{N}-\overset{⌣}{N}\right)$$

where $\overset{⌣}{b}$ and $\hat{b}$ are the position estimators of the fixed and float solution respectively, $\hat{N}$ is the float ambiguity vector with the variance-covariance matrix $Q_{\hat{N}}$, $\overset{⌣}{N}$ is the integer ambiguity vector and $Q_{\hat{b}\hat{N}}$ is the covariance matrix of $\hat{b}$ and $\hat{N}$.

It should be noted that the main difficulty for PPP AR is to fix the narrow-lane ambiguity reliably. This paper studies only the narrow-lane ambiguity resolution, therefore, in the interest of brevity, the phrase “narrow-lane ambiguity” will be simply referred to by the word “ambiguity” throughout the rest of this article if there is no additional explanation. Acceptance of an incorrect integer will lead to an unacceptable positioning error, so the user should carefully validate integer ambiguities. The success rate and ratio-test value are often used for ambiguity validation once ambiguity resolution is completed [23,24]. The success rate is a model-driven index, which indicates the strength of the underlying model and assumption of the variance of measurement noises, and gives a quantitative assessment of the probability of correct fixing. The bootstrapped success rate which has been proved to be a good approximation of the actual integer least squares (ILS) success rate is used here. It can be calculated with the following formula [25]:

(2) $$P=\prod_{i=1}^{n}\left(2\text{Φ}\left(\frac{1}{2{\text{σ}}_{{\hat{N}}_{i|I}}}\right)-1\right)$$

with:

(3) $$\text{Φ}\left(x\right)=\frac{1}{\sqrt{2\pi }}\int_{-\infty }^{x}e^{-t^{2}/2}dt$$

where the shorthand notation ${\hat{N}}_{i|I}$ stands for the ith least squares ambiguity obtained through a conditioning on the previous I={1,…,(i−1)} sequentially rounded ambiguities and σ its standard deviation. This model-driven approach is simple and valid, since the failure rate can be determined prior to the actual integer estimation step. In addition, an overall and rigorous quality description of the fixed solution is available [26]. However, the success rate is not directly dependent upon the actual measurements. Any undetected biases in the actual measurements may affect the real success rate although the computed success rate could still be high. Therefore one cannot simply decide to accept the integer solution only relying on the success rate with sufficient confidence. In practice, the ratio-test, defined as the ratio of the second minimum quadratic form of the residuals to the minimum quadratic form of the residuals, is a data-driven index of the reliability of AR and frequently applied to assess the closeness of the float solution to its nearest integer vector [27]. It is given by:

(4) $$R=\frac{{\left\|\hat{N}-{\overset{⌣}{N}}_{2}\right\|}_{Q_{\hat{N}}}^{2}}{{\left\|\hat{N}-\overset{⌣}{N}\right\|}_{Q_{\hat{N}}}^{2}}$$

where ${\overset{⌣}{N}}_{2}$ is the second-best integer ambiguity solution. In practice it often works satisfactorily with a fixed threshold [23]. However, the overall quality of the fixed solution cannot be properly estimated by ratio test [28]. Therefore, the ambiguity validation does not always work reliably with any single validation test above. Thus, we applied both the well-known ratio test and success rate to validate the ambiguities for both FAR and PAR.

### 2.2. A Modified Partial Ambiguity Resolution Method for the PPP User

Instead of fixing all ambiguities for PPP, a modified PAR method is used to find a subset of integers which can be fixed with high confidence if FAR is unsuccessful. Like the method proposed by Wang and Feng [5], our modified PAR method combines both the success rate and the ratio test for ambiguity validation.

In addition, decorrelated ambiguities rather than the raw float ambiguities are used to determine the subset. This is different from the method used by Shi and Gao [16], in which they used the un-decorrelated ambiguities. In our method, the potential ambiguity subset is validated by both the success rate and ratio-test in an iterative process, so that an ambiguity subset with high probability of correct fixing may be found. 

**Figure 1:**
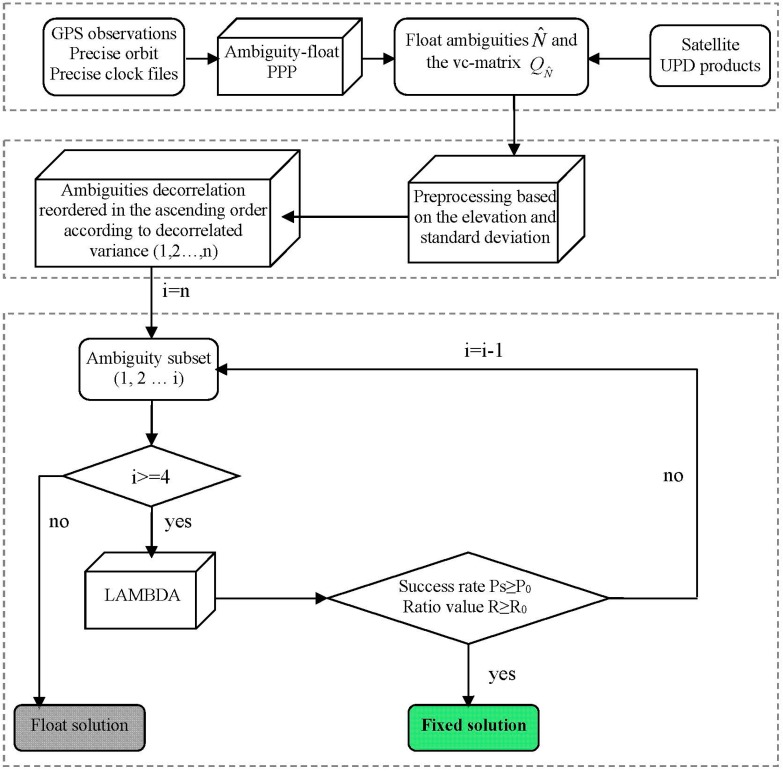
A flowchart of partial ambiguity resolution with predefined success rate and ratio value.

This method is also different from the WF method in which the largest subset with a success rate above the threshold is first determined and then validated by the ratio-test. In their approach, they ignored that it is still possible to potentially find a smaller subset which can pass the ratio test even when their defined subset fails to pass the ratio test. Hence, our method is likely to increase the possibility of finding a potential subset of decorrelated ambiguities.

A flowchart of our PAR procedure is shown in Figure 1. First, no attempt is made to fix an ambiguity if the elevation of that satellite is smaller than a threshold (10 degrees) or the standard deviation of the ambiguity is larger than a predefined threshold (1.0 cycle) in the preprocessing stage. This is because a newly rising satellite’s ambiguity or a re-initialization of one following a cycle slip or data interruption is too noisy to be correctly fixed. The threshold is empirically chosen to primarily eliminate the ambiguity which has a low-precision and then accelerate the ambiguity subset determination in the following steps. Second, all valid ambiguities after the first step are decorrelated and reordered in the ascending order according to their decorrelated variances. Third, the LAMBDA method is used to fix the ambiguities of the selected subset. Fourth, the bootstrapped success rate *P* and the ratio value *R* are calculated. If *P* < *P*_0_ (predefined) or *R* < *R*_0_ (predefined), the last ambiguity with lowest-precision is rejected, and then the third step is repeated, unless the number of remaining ambiguity candidates is fewer than four, when only the float solutions are available. Otherwise, the integer ambiguities which have satisfied both the requirement of the success rate and the ratio test are accepted and the procedure ends.

## 3. Experimental Section

**Figure 2:**
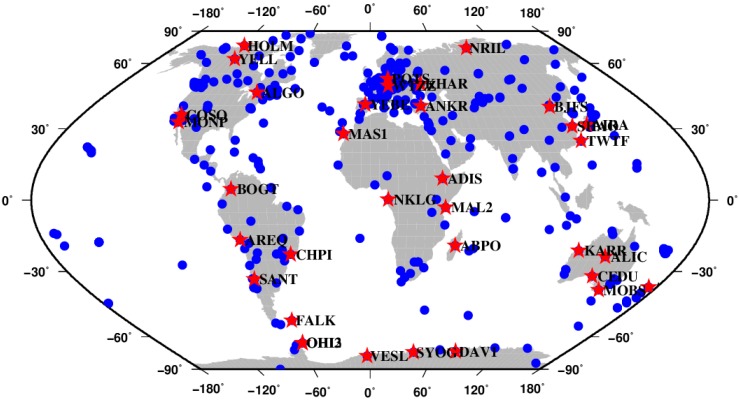
Distribution of the global reference network and user stations. The blue dots are the reference stations used for UPDs estimation; the red stars are the user stations in the test. This figure is created with the GMT software by Wessel and Smith [37].

To assess the performance of the proposed PAR algorithm, about 420 IGS stations are used as the reference network to generate the wide-lane and narrow-lane UPDs corrections for all healthy satellites. The remaining 35 stations which are located in seven continents are used as test user stations. These user stations are approximately uniformly distributed along the geographical latitude and longitude, as shown in Figure 2. Daily GNSS measurements from those IGS stations observed during DOY 021 to 027 in 2012 are used in this study. At the server end, the SD wide-lane UPDs are estimated every day, while the SD narrow-lane UPDs are estimated every 15 min. In order to eliminate the rank deficiency in UPD estimation, one arbitrarily selected UPD must be set to zero. One of the satellite UPDs was chosen in our study because that the satellite UPD is more stable than the receiver one [29]. Then the global UPD products referenced to that satellite can be estimated. We randomly choose PRN 2 as a reference satellite for SD wide-lane and narrow-lane UPDs estimation. Of course, any other healthy GPS satellite can also be used as a reference one. The SD wide-lane and narrow-lane UPDs of all satellites are estimated referenced to PRN 2, which is healthy [29]. At the user end, daily observations are separated into eight 3-hour-long observation segments for experiments. The processing results of the first 90 min of observations are mainly used for analyzing the TTFF, while the results of the second 90 min of observations are mainly used for analyzing the fixing rate. Both static and kinematic experiments are conducted to analyze the performance of the proposed PAR method.

We use the final products of the satellite orbits and clocks and the differential code biases produced by Center for Orbit Determination in Europe (CODE) [30], and apply the absolute antenna phase centers model [31], the phase wind-up corrections [32]. The true coordinate benchmarks are from IGS weekly solutions. For troposphere delay estimation, the zenith dry component of tropospheric delays can be corrected with the priori Saastamoinen model [33]. The zenith wet delay (ZWD) is estimated as an unknown parameter. The global mapping function [34] is used to project the slant hydrostatic and wet delays to the zenith delays. The troposphere gradient parameters are sometimes estimated in high-precision scientific software, to account for the bulk of the asymmetric delay. However, as reported by [35], these gradients account for only a single main direction of asymmetry, and their estimation reduces the redundancy of the solution. For the sake of simplicity, the estimation of horizontal troposphere gradients is not considered in this study. All the observations are weighted according to their elevation and the elevation cut-off angle for data is set to 7 degrees. The parameters including positions, ZWD and ambiguities are estimated in a Kalman filter. For the Kalman filter, the spectral density value for the ZWD parameter is empirically set to 10^−^^8^ m^2^/s. The ambiguity parameters and static position coordinates are considered as constants. But in kinematic mode, the kinematic position coordinates are modeled as white noise. The initial standard deviation values for GPS phase and code observations at zenith direction are set to 0.003 and 0.3 m, respectively. The satellite with good quality observations and the highest elevation angle is selected as the reference satellite for PPP AR. In the ambiguity resolution processing, a single ambiguity with elevation angle lower than 10 degrees or the standard deviation larger than 1.0 cycle will be eliminated in the preprocessing stage. The critical criterion of the ratio value and success rate is selected as 2 and 0.99 respectively. After successful ambiguity resolution, we also use the “fix and hold” mode proposed by Takasu and Yasuda [36] which is a tight constraint on fixed ambiguities. The constraint is applied as a “pseudo”-measurement update, which treats the SD integer ambiguities as observation of the ambiguity parameters in the filter state. At first, estimated float ambiguities are resolved by the usual way but once the integer solutions are verified by the validation process, the tight constraint to the integer solutions is introduced into the next update of the filter. A fixed ambiguity is held to an integer value until a cycle-slip occurs or the filter diverged with large residuals.

## 4. Results and Discussion

In total about 1960 tests were used. The effectiveness of the modified PAR method is first assessed by comparing the possibility of successfully finding a potential subset using the modified PAR method and the WF method. Then, the test data is processed using traditional PPP FAR and the proposed PPP PAR method, respectively. The results using two processing methods are analyzed to assess the performance of our modified PPP PAR methods, including the TTFF, fixing rate and positioning accuracy.

### 4.1. Comparison of the Probability of Successful Fixing

The ambiguity subset determination is the key to the PAR algorithm. Different from the WF method in which the largest subset with a success rate above the threshold is determined first and then validated by the ratio-test, in our modified method, the success rate and ratio-test are simultaneously used in an iterative process to find a potential subset. Therefore, even if their determined subset fails to pass the ratio test, in theory, it is still possible to potentially find a smaller subset which has a higher success rate and can pass the ratio test in our method.

All the 3-hour-long observations on DOY 021 in 2012 are processed using these two PAR methods, in static PPP mode. And then the probability of successful fixing is compared. It should be noted that the same minimum elevation and success rate threshold are also applied in the WF method, to make the two results comparable. Table 1 gives the number of successful and unsuccessful fixing epochs obtained by two methods. Using the WF method, 84.1% of epochs can achieve a fixed solution. Still there are about 14,000 epochs in which the determined largest subset has a success rate above the threshold, but where the ratio test fails. However, after utilizing our modified PAR method, the percentage of the successful fixing epochs obviously increases to 99.1%. It demonstrates that our modified PAR method can increase the possibility of finding a potential subset of decorrelated ambiguities which can be fixed with high confidence.

**Table 1:** The number of successful and unsuccessful fixing epochs obtained by WF and our PAR methods.

PAR Method	Successful Fixing	Unsuccessful Fixing
WF	74,018	13,955
Our	87,195	778

### 4.2. Comparison of the Time to First Fix

**Figure 3:**
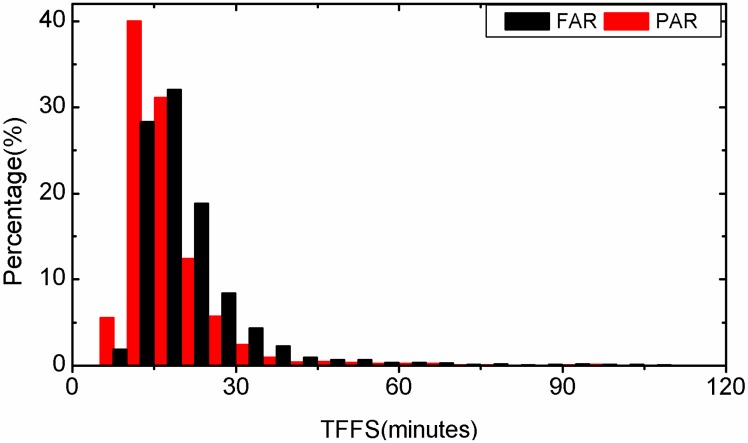
Distribution of TTFF of static PPP by employing FAR and PAR, respectively.

**Figure 4:**
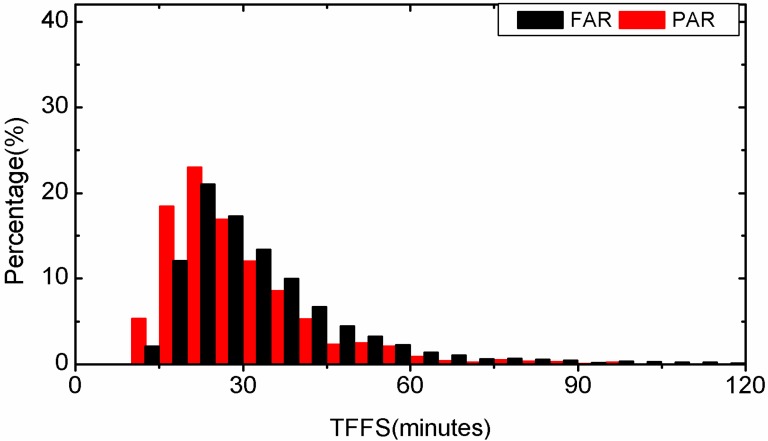
Distribution of TTFF of kinematic PPP by employing FAR and PAR, respectively.

First, the forward-Kalman-filter PPP is applied to all 3-hour-long observations with FAR and PAR, respectively. The TTFF of each solution is recorded. Statistical results of the TTFF for all observations are plotted in Figure 3 for static PPP and in Figure 4 for kinematic PPP. In static PPP, the average TTFF is 20.1 min for FAR while the PAR method needs only 17.1 min. The average TTFF is reduced by 14.9% with PAR. In kinematic PPP, the FAR method needs 33.8 min, but for PAR 28.7 min are enough. The average TTFF is reduced by 15.1% with PAR. In addition, we find that in static PPP, 59.9% of the examples have a shorter TTFF by using the PAR method, and in kinematic PPP, the percentage of the instances which have a shorter TTFF by using the PAR method is 57.6%. In those cases, the TTFF of PAR has apparently outperformed that of FAR. This is probably because some ambiguities are biased or have a low-precision and decrease the performance of FAR. Nevertheless, the problematic ambiguity or ambiguities can be identified and removed in the PAR method. Therefore, a successful AR can still be achieved. In other cases, the PAR method has almost the same performance as FAR. It is usually true for the cases that FAR can be implemented with observations of high quality and no ambiguity would be rejected in the PAR procedure.

**Figure 5:**
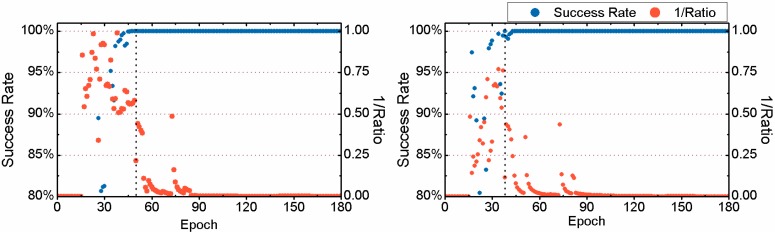
Success rate and reciprocal of the ratio test value for for static PPP for the observations of the 6th session from MAS1 on 24 January 2012, achieved by FAR (**Left**) and PAR (**Right**), respectively.

**Figure 6:**
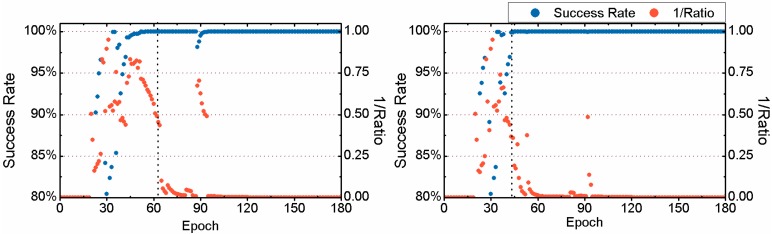
Success rate and reciprocal of the ratio test value for kinematic PPP for the observations of the 6th session from MAS1 on 24 January 2012, achieved by FAR (**Left**) and PAR (**Right**), respectively.

Here we take the 3-hour-long observation of the 6th session from the MAS1 station on 24 January 2012 as a typical example to compare the TTFF between FAR and PAR. Details are given in Figure 5 and Figure 6. It should be noted that the reciprocal of the ratio test value given by Equation (4) is plotted in the two figures for a clear display. Comparing the two sub-figures of Figure 5, we can find that 24.5 min are required to achieve the first fixed solution for FAR, while only 18.5 min are needed for PAR. The TTFF of static PPP is reduced by 6 min. In addition, in the case of FAR, before the first fixed solution, the success rate of some epochs can be acceptable, but the ratio value is usually less than 2 which indicates an unreliable fixed solution. At those epochs, both the success rate and ratio value increase significantly after using the PAR method. The corresponding results for kinematic PPP are shown in Figure 6. We can see that both the success rate and ratio value have been improved by PAR, during the period between the 43th and 62th epoch. For FAR, 30.5 min are required to achieve the first fixed solution while for PAR only 21.0 min are needed. The TTFF of this example is reduced by 9.5 min.

### 4.3. Fixing Rate Comparison

**Figure 7:**
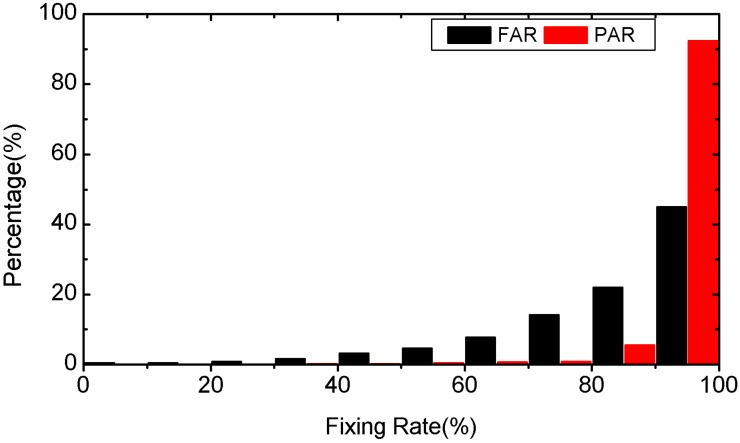
Distribution of fixing rate percentages of static PPP by employing FAR and PAR, respectively.

**Figure 8:**
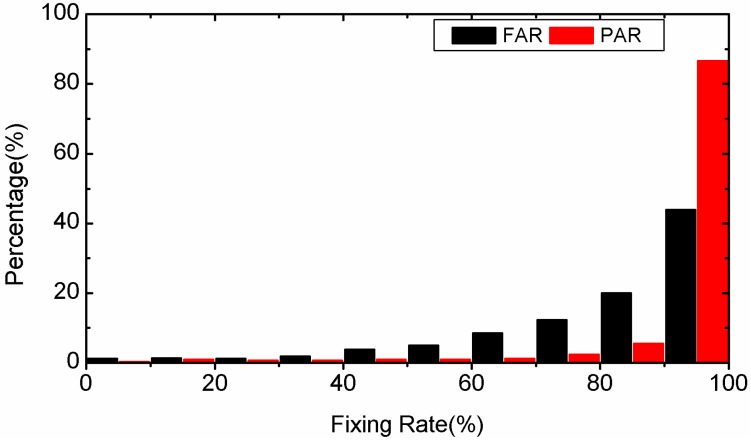
Distribution of fixing rate percentages of kinematic PPP by employing FAR and PAR, respectively.

In this section, we will compare the fixing rate of FAR and PAR using the results of the second 90 min of each 3-hour-long observations. The statistical results for static PPP are plotted in Figure 7 and those for kinematic PPP are presented in Figure 8. Compared to FAR, the average fixing rate of PAR is added by 14.7% (from 83.5% to 98.2%) in static PPP and by 15.1% (from 80.1% to 95.2%) in kinematic PPP. This can be explained by the fact that when FAR fails, a successful ambiguity resolution is still possible with PAR after removing the bad quality ambiguity. In addition, for both FAR and PAR, the fixing rate in kinematic PPP is lower than in static PPP. This is because the float ambiguity precision in kinematic PPP is lower than in static PPP.

**Figure 9:**
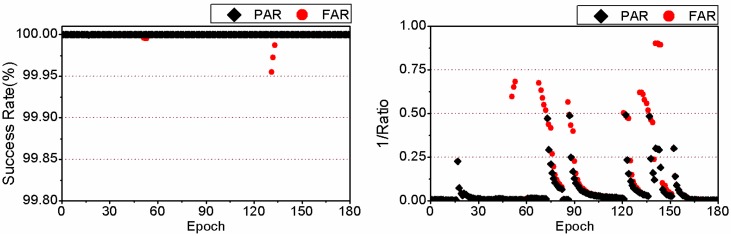
Success rate (**Left**) and reciprocal of ratio test value (**Right**) of static PPP with PAR and FAR for the observations of the 4th session of the MOBS station on 25 January 2012.

**Figure 10:**
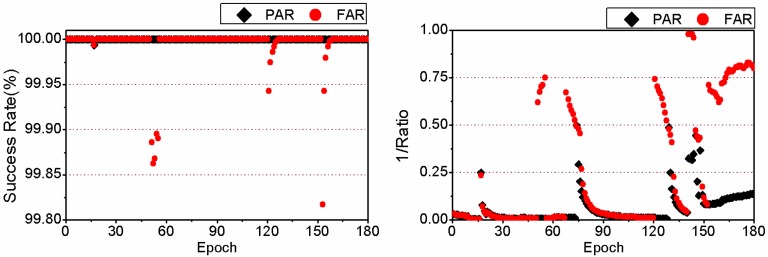
Success rate (**Left**) and reciprocal of ratio test value (**Right**) of kinematic PPP with PAR and FAR for the observations of the 4th session of the MOBS station on 25 January 2012.

We take another 3-hour-long observation of the 4th session of the MOBS station on 25 January 2012 as a typical example to compare the success rate and ratio test value between PAR and FAR in detail. The success rates for static PPP are shown in the left sub-figure of Figure 9. We can find that the success rates of PAR are always equal to or higher than those of FAR. The reciprocal of the ratio value series of static PPP are given in the right-hand sub-figure of Figure 9. The reciprocal of the ratio test value for PAR are all lower than the threshold 0.5. However, there are quite a few epochs in which the reciprocal of the ratio are larger than 0.5 for FAR. In those cases, the optimal integer estimators of the ambiguities cannot be distinguished reliably from the sub-optimal ones. As a whole, the fixing rate is improved from 81.1% for FAR to 100.0% for PAR. The success rate series and the reciprocal of ratio series of kinematic PPP are shown in Figure 10. As the figures show, the success rate of PPP AR can be improved by PAR significantly compared to FAR. There are many epochs whose ratio test value is less than the threshold for FAR, but there is no epoch whose ratio test value is less than the threshold of 2 for PAR.

### 4.4. Positioning Accuracy Comparison

In this part, the positioning accuracy of the PAR and FAR solutions are compared. We have calculated the root mean square (RMS) values of the positioning biases over all sessions. For static PPP, the difference of average RMS between FAR and PAR solutions is only 0.05, −0.03 and −0.21 mm in the east, north and up directions, respectively. The difference is not obvious because that the model strength of static PPP is strong enough. The average RMS of PAR solutions is 7.2, 6.3 and 18.4 mm in the east, north and up directions, respectively.

**Figure 11:**
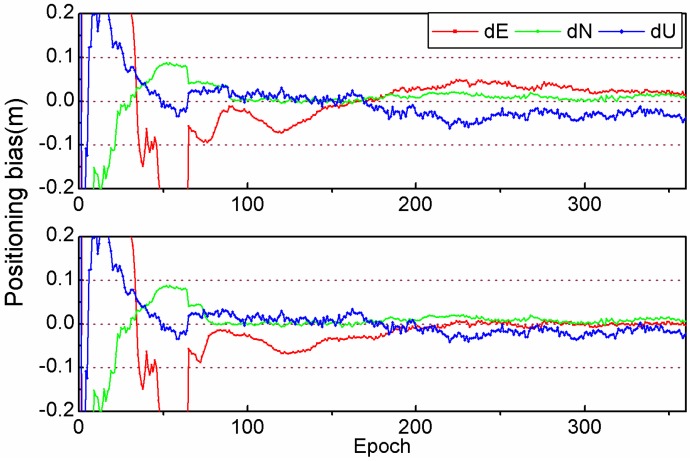
Coordinate biases of kinematic PPP for the observations of the 2nd session from station SANT on 26 January 2012, with respect to the coordinate benchmarks from IGS weekly solutions, with the FAR (**Top**) and PAR (**Bottom**) method, respectively.

For kinematic PPP, we first take the 3-hour-long observation of the 2nd session from SANT station on 26 January 2012 as a representative example to analyze the impact of PAR on the positioning accuracy. The epoch-wise coordinate biases in three directions are plotted in Figure 11. From the 88th to 185th epochs (the interval is 30 s), the fixed solution can be achieved successfully by FAR and PAR. The PAR and FAR solutions are close to each other, and the positioning biases are a few centimeters. However, for the later epochs where often only a float solution can be achieved by FAR, we see that the coordinate biases are obviously larger than those of the PAR solution in which partial ambiguities could be fixed continuously. The fixed solution can be achieved accurately for all epochs when the PAR method is employed.

**Figure 12:**
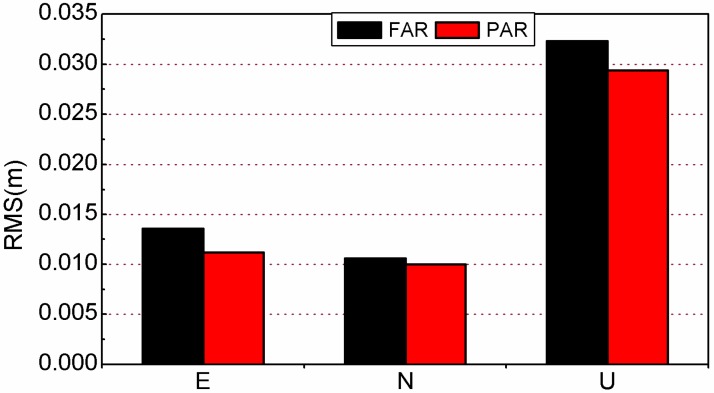
The average RMS of station position in east, north and up directions for kinematic PPP with the FAR and PAR methods.

We have calculated the average RMS of all test sessions (see Figure 12). The RMS can be reduced by 17.6%, 5.7% and 9.0% (from 13.6, 10.6 and 32.3mm to 11.2, 10.0 and 29.4 mm in east, north and vertical) when the PAR method is applied to the same data set. It should be noted that the accuracy of the east component is considerably worse than that of the north component for FAR. The reason is that the integer ambiguity cannot be correctly resolved in some epochs, which results in a float solution. Generally speaking, the accuracy of the east component is lower than that of north component for a PPP float solution [7]. 

Nevertheless, once the PAR method is applied, the accuracy of the east component can be improved so that it is comparable with that of the north component. In theory, the position benefits much more with more correctly fixed ambiguities. If FAR can be correctly performed, the positioning accuracy of a single epoch should be a little better than that from PAR. Nevertheless, as discussed above, FAR suffers from slower TTFF and lower fixing rate than PAR. In the case FAR fails while PAR succeeds, the position accuracy from FAR solutions would be much worse than that from PAR solutions. As a composite result, compared with FAR, the positioning accuracy of kinematic PPP is obviously improved by our PAR strategy.

## 5. Conclusions

Compared with short-baseline relative positioning, it is more difficult to fix all ambiguities reliably for PPP. We propose a modified PAR method for GPS PPP to determine a potential ambiguity subset when full ambiguity resolution fails. Different from the WF method in which the largest subset with a success rate above the threshold is determined and then validated by the ratio-test, in our modified method, the success rate and ratio-test are simultaneously used in an iterative process to find a potential subset. Our modified PAR method can increase the possibility of finding a potential subset of decorrelated ambiguities because that when the subset from conventional method fails to pass the ratio test, it is still possible to potentially find a smaller subset which has a higher success rate and can pass the ratio test in our method.

GPS measurements of about 450 stations from the IGS network during DOY 021 to 027 in 2012 are used in this experiment. Numerous experimental results show that the PAR method outperforms FAR. This is because, when FAR fails, successful PAR is still possible when bad-quality ambiguities are removed. In static PPP, the average TTFF can be reduced by 14.9%, from 20.1 min for FAR to 17.1 min for PAR. In kinematic PPP, the average TTFF is reduced by 15.1% from 33.8 min for FAR to 28.7 min for PAR. The average fixing rate of PAR is increased from 83.5% to 98.2% in static PPP and from 80.1% to 95.2% in kinematic PPP. For static 3-hour-long PPP, the static positioning accuracy of FAR and PAR have little difference due to the strong model strength of static PPP. However, for kinematic PPP, the RMS can be reduced by 17.6%, 5.7% and 9.0% (from 13.6, 10.6 and 32.3 mm to 11.2, 10.0 and 29.4 mm) in north, east and vertical, when the PAR method is applied for the same data set. We demonstrated that our PAR method can improve the performance of GPS PPP with dual-frequency observations, including speeding up the first fixed solution, increasing the fixing rate, and improving the positioning accuracy of kinematic PPP.

## References

[1] Teunissen P.J.G., Joosten P., Tiberius C. Geometry-free ambiguity success rates in case of partial fixing. Proceedings of the ION NTM.

[2] Cao W., O’Keefe K., Cannon M. Partial ambiguity fixing within multiple frequencies and systems. Proceedings of the ION GNSS.

[3] Henkel P., Günther C. (2010). Partial integer decorrelation: Optimum trade-off between variance reduction and bias amplification. J. Geod..

[4] Parkins A. (2011). Increasing GNSS RTK availability with a new single-epoch batch partial ambiguity resolution algorithm. GPS Solut..

[5] Wang J., Feng Y. (2013). Reliability of partial ambiguity fixing with multiple GNSS constellations. J. Geod..

[6] Zumberge J.F., Heflin M.B., Jefferson D.C., Watkins M.M., Webb F.H. (1997). Precise point positioning for the efficient and robust analysis of GPS data from large networks. J. Geophys. Res..

[7] Ge M., Gendt G., Rothacher M., Shi C., Liu J. (2008). Resolution of GPS carrier-phase ambiguities in Precise Point Positioning (PPP) with daily observations. J. Geod..

[8] Laurichesse D., Mercier F., Berthias J.P., Broca P., Cerri L. (2009). Integer ambiguity resolution on undifferenced GPS phase measurements and its application to PPP and satellite precise orbit determination. Navigation.

[9] Bertiger W., Desai S., Haines B., Harvey N., Moore A., Owen S., Weiss J. (2010). Single receiver phase ambiguity resolution with GPS data. J. Geod..

[10] Teunissen P.J.G., Odijk D., Zhang B. (2010). PPP-RTK: Results of CORS network-based PPP with integer ambiguity resolution. J. Aeronaut. Astronaut. Aviat. Ser. A.

[11] Collins P., Bisnath S., Lahaye F., Héroux P. (2010). Undifferenced GPS ambiguity resolution using the decoupled clock model and ambiguity datum fixing. Navigation.

[12] Loyer S., Perosanz F., Mercier F., Capdeville H., Marty J. (2012). Zero-difference GPS ambiguity resolution at CNES-CLS IGS Analysis Center. J. Geod..

[13] Zhang X., Li P., Guo F. (2013). Ambiguity resolution in precise point positioning with hourly data for global single receiver. Adv. Space Res..

[14] Geng J., Teferle F.N., Meng X., Dodson A.H. (2011). Towards PPP-RTK: Ambiguity resolution in real-time precise point positioning. Adv. Space Res..

[15] Verhagen S., Teunissen P.J.G., van der Marel H., Li B. GNSS ambiguity resolution: Which subset to fix?. Proceedings of the 2011 IGNSS Symposium, International Global Navigation Satellite Systems Society.

[16] Shi J., Gao Y. A fast integer ambiguity resolution method for PPP. Proceedings of the ION GNSS.

[17] Dow J.M., Neilan R.E., Rizos C. (2009). The international GNSS service in a changing landscape of global navigation satellite systems. J. Geod..

[18] Blewitt G. (1989). Carrier phase ambiguity resolution for the Global Positioning System applied to geodetic baselines up to 2000 km. J. Geophys. Res..

[19] Li X., Zhang X. (2012). Improving the estimation of uncalibrated fractional phase offsets for PPP ambiguity resolution. J. Navig..

[20] Melbourne W.G. The case for ranging in GPS-based geodetic systems. Proceedings of the First International Symposium on Precise Positioning with the Global Positioning System.

[21] Wei M., Schwarz K.P. Fast ambiguity resolution using an integer nonlinear programming method. Proceedings of the ION GNSS.

[22] Teunissen P.J.G. (1995). The least-squares ambiguity decorrelation adjustment: A method for fast GPS integer ambiguity estimation. J. Geod..

[23] Ji S., Chen W., Ding X., Chen Y., Zhao C., Hu C. (2010). Ambiguity validation with combined ratio test and ellipsoidal integer aperture estimator. J. Geod..

[24] Verhagen S. (2005). On the reliability of integer ambiguity resolution. Navigation.

[25] Teunissen P.J.G. (1998). Success probability of integer GPS ambiguity rounding and bootstrapping. J. Geod..

[26] Teunissen P.J.G., Verhagen S. GNSS Ambiguity Resolution: When and How to Fix or not to Fix?. Proceedings of the VI Hotine-Marussi Symposium of Theoretical and Computational Geodesy: Challenge and Role of Modern Geodesy.

[27] Frei E., Beutler G. (1990). Rapid static positioning based on the fast ambiguity resolution approach FARA: Theory and first results. Manuscr. Geod..

[28] Teunissen P.J.G., Verhagen S. (2009). The GNSS ambiguity ratio-test revisited: A better way of using it. Surv. Rev..

[29] Zhang X., Li P. (2013). Assessment of correct fixing rate for precise point positioning ambiguity resolution on global scale. J. Geod..

[30] Dach R., Brockmann E., Schaer S., Beutler G., Meindl M., Prange L., Bock H., Jaggi A., Ostini L. (2009). GNSS processing at CODE: Status report. J. Geod..

[31] Schmid R., Rothacher M., Thaller D., Steigenberger P. (2009). Absolute phase center corrections of satellite and receiver antennas. GPS Solut..

[32] Wu J.T., Wu S.C., Hajj G.A., Bertiger W.I., Lichten S.M. (1993). Effects of antenna orientation on GPS carrier phase. Manuscr. Geod..

[33] Saastamoinen J., Henriksen S., Mancini A., Chovitz B. (1972). Atmospheric correction for troposphere and stratosphere in radio ranging of satellites. the Use of Artificial Satellites for Geodesy, Geophysics Monograph Series.

[34] Boehm J., Niell A., Tregoning P., Schuh H. (2006). Global Mapping Functions (GMF): A new empirical mapping function based on numerical weather model data. Geophys. Res. Lett..

[35] Urquhart L., Nievinski F.G., Santos M. (2012). Ray-traced slant factors for mitigating the tropospheric delay at the observation level. J. Geod..

[36] Takasu T., Yasuda A. Kalman-filter-based integer ambiguity resolution strategy for long-baseline RTK with ionosphere and troposphere estimation. Proceedings of the ION GNSS.

[37] Wessel P., Smith W.H.F. (1998). New, improved version of generic mapping tools released. EOS Trans. AGU.

